# Delivery of *B. subtilis* into Animal Intestine Using Chitosan-Derived Bioresorbable Gel Carrier: Preliminary Results

**DOI:** 10.3390/gels9020120

**Published:** 2023-02-01

**Authors:** Alexander Samokhin, Anastasia Korel, Elena Blinova, Alexander Pestov, Galina Kalmykova, Nadezhda Akulova, Viktoria Betz, Vadim Tkachenko, Ekaterina Litvinova

**Affiliations:** 1 Novosibirsk State Technical University, 630073 Novosibirsk, Russia; 2 Institute of Organic Synthesis n.a. I. Ya. Postovsky UB RAS, 620137 Ekaterinburg, Russia; 3 Institute of Nuclear Physics SB RAS, 630090 Novosibirsk, Russia; 4 Scientific Research Institute of Neurosciences and Medicine SB RAS, 630117 Novosibirsk, Russia

**Keywords:** bacteria, bacillus subtilis, intestine, carrier, sodium N-(2-sulfonatoethyl)chitosan, gel, release, probiotics, cytotoxicity, animal

## Abstract

The oral delivery of bacteria in the human intestine is of great interest because of its potential to correct the gut microbiota and treat inflammatory bowel diseases. The aim of this study was to evaluate sodium N-(2-sulfonatoethyl)chitosan gel cross-linked with glutaraldehyde as a delivery carrier for probiotic bacteria to the gut using in vitro and in vivo experiments. The bacterial test strain was *B. subtilis 20*. The cytotoxicity of the gel was evaluated via cell culture using flow cytometry and light microscopy. The gel as a delivery system was assessed by the dye release in medium with different pH levels in vitro, and by bacterial titer monitoring in mouse feces using the microbiology method in vivo. Results of an in vitro experiment showed that tested gel has no cytotoxicity. The use of gel as a carrier for bacterial delivery into the intestine was more effective than oral gavage of bacterial suspension. Therefore, gel delivery of bacteria decreased the titer level by up to two times. However, a gavage of bacterial suspension decreased the titer level by over 200 times. Tested gel has the potential to be a carrier for the safe delivery of bacteria to the intestine through the stomach, reducing the rate of the elimination of probiotic bacteria from the intestine.

## 1. Introduction

Numerous articles about microbiota–host interactions published in the past two decades have confirmed that the gut microbiota has a significant impact on host homeostasis. There is interest in ways to correct the imbalance in the microbiota to treat intestinal inflammation [1].

The pathogenesis of inflammatory bowel diseases (IBD) related to microbiota occurs in both animals and humans. The microbiota plays important roles in the pathophysiology of IBD by impacting the immune system, the host metabolism and gastrointestinal function [1,2,3].

The treatment of IBD involves restoring the microbiota by first decreasing the amount of existing pathobionts and then delivering probiotics, therapeutic agents, or microbiota of from a healthy donor. There are two ways to deliver bacteria in IBD patients: transoral delivery and fecal transplantation. Both ways have their drawbacks. For transoral delivery, the beneficial bacteria need to be protected from damage by the acidic environment of the stomach [4,5].

The *Bacillus* species are probiotic bacteria that support a healthy gut microbiome and promote digestion and immunity. They include *B. subtilis*, *B. coagulans, B. licheniformis* and *B. clausii* [6,7,8]. However, the lowest favorable pH for the *B. subtilis* strains is 5.0; furthermore, this bacterium prefers neutral pH environments [9,10,11]. An optimal pH is crucial in order for the bacteria to survive during the stomach passage, which has an aggressively acidic pH, until it reaches the treatment site.

Different delivery systems for bacterial safety are used to avoid their destruction in acidic pHs, including biodegradable gel carriers based on alginate, chitosan or gelatin [12,13,14,15]. At the same time, the carrier should provide not only protection for the bacteria from the gastric acid, but also should be able to effectively release it when it reaches the alkaline pH of the intestine.

Despite there being a wide selection of delivery systems for all proven probiotics (*Lactobacillus, Bifidobacterium, Bacillus, Streptococcus* and others), most studies are still at the in vitro stage. Carriers require bacteria delivery capabilities to be tested in vivo [15].

It is known that sulfated polysaccharides are ubiquitous in living systems. These polysaccharides are involved in such biological functions as organism development, cell proliferation and differentiation, tissue homeostasis and host defense. Engineered sulfated polysaccharides are structural derivatives not found in nature. They are generated through the chemical and enzymatic modification of natural polysaccharides, as well as chemically synthesized oligo- and polysaccharides. Engineered sulfated polysaccharides exhibit novel and augmented biological properties compared with their unmodified counterparts, mainly through facilitating interactions with other macromolecules [16]. However, data on the usage of carrier gels made from such modified sulfated chitosan in living systems to deliver bacteria into the gut are scarce.

In this paper, we present a short communication of our pilot study, which aimed to identify the properties of a bioresorbable sodium N-(2-sulfonatoethyl)chitosan (SEC) gel crosslinked with glutaraldehyde as a potential carrier for the oral delivery of probiotics through the gastrointestinal tract in an animal model using *B. subtilis 20* as a test strain.

## 2. Results

### 2.1. Release of Dye from the Gel in Acidic and Alkaline Media

**Figure 1:**
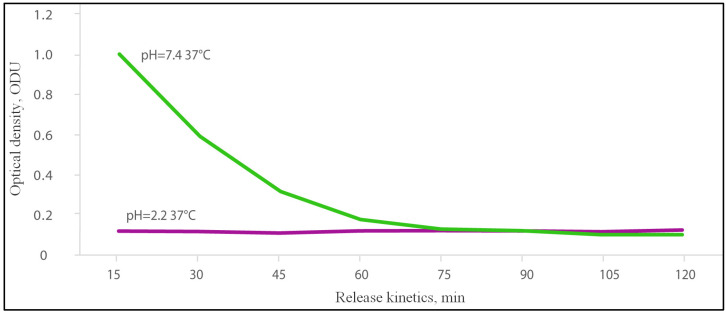
Kinetics of dye release from the sodium N-(2-sulfonatoethyl)chitosan gel in two different pH mediums at 37 °C.

In an acidic medium (pH = 2.2), the dye was not released from the SEC gel. The level of dye was constantly in a range of 0.098–0.106 optical density units (ODU). However, the dye was successfully released from the SEC gel in the alkaline medium (pH = 7.4). There was a more effective release in the first 15 min, which amounted to 1.008 ODU, followed by a slow release of dye until reaching the lower limit of detection: 0.595 ODU at 30 min, 0.321 ODU at 45 min, 0.181 ODU at 60 min and 0.103 at 105 min (Figure 1).

### 2.2. Cell Viability/Cytotoxicity of Fibroblast Culture with Gel

**Figure 2:**
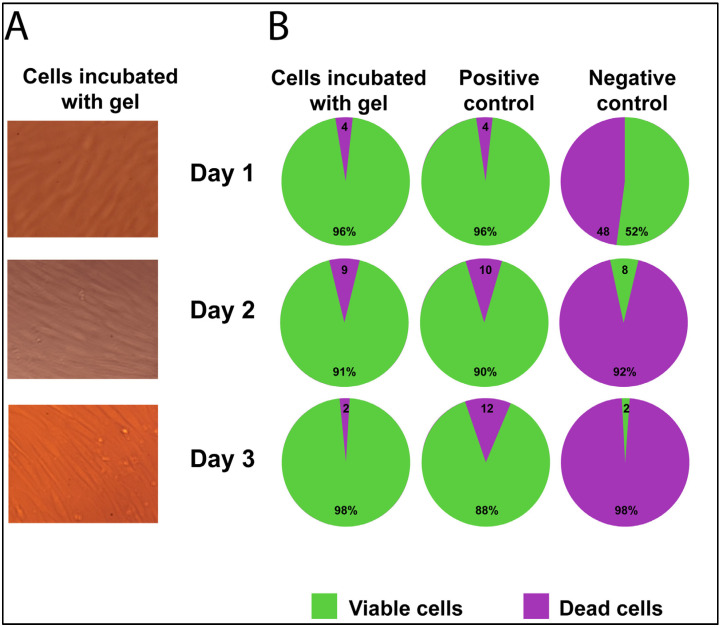
Cell viability/cytotoxicity of fibroblasts, observed during three days of culture with the gel. (**A**). Cell culture microscopy images. (**B**). Live/dead cells ratio (flow cytometry with Calcein-AM and 7-AAD dyes).

Microscopy of the cell culture showed no signs of cytotoxicity after incubation with the gel sample: fibroblasts had a typical morphology and proliferated and formed a monolayer for three days. There were no rounded cells that are specific for dead cells (Figure 2A).

The percent of viable cells on Day 1 did not differ from the positive control—it was 96% in both groups (Fisher exact test = 1.0). The percent of viable cells in the negative control (with DMSO) was two times lower than in the positive control (48% vs. 96%) (Chi-square = 57.1; *p* < 0.00001).

On Day 2, 91% of the cells incubated with gel were alive, and 90% were still living in the positive control, which was almost equal to the percentage on Day 1 (Chi-square = 2.1 and Chi-square = 2.8, *p* > 0.05, respectively). However, in the negative control, the number of live cells on Day 2 was reduced drastically to 8% (Chi-square = 39.7; *p* < 0.0001).

On Day 3, there was no significant decrease in the number of live cells compared to Day 2 in the positive control—88% (Chi-square = 0.2; *p* > 0.05). In the negative control, the number of live cells decreased by four times to 2% (Chi-square = 3.8; *p* = 0.05). In the cells incubate with gel, after three days, the number of live cells was increased to 98% in comparison with Day 2’s values (Chi-square = 4.7; *p* = 0.03).

### 2.3. Test Strain of B. subtillus 20 Transit in the Gastrointestinal Tract of the Mice

Microbiological analysis of the feces of mice fed a standard diet showed the absence of the test strain *B. subtillus 20* on Day 0. After five days of administering the gel loaded with test strain *B. subtillus 20* during feeding, the number of bacteria increased, and the number was only two times less than in the loaded gel (baseline titer). After the gel additive was withdrawn on Day 5, the number of bacteria decreased by 10 and 40 times on Days 6 and 7, respectively (Table 1).

At the same time, the titer of the test strain *B. subtilis 20* in the feces of mice fed a diet with an additive suspension of *B. subtilis 20* (concentration of 10^9^ spores/g of diet) decreased by more than 200 times during feeding.

## 3. Discussion

The use of synthetic sulfated derivatives of chitosan as carriers has been discussed in various articles over the last five years. Basically, these materials are described as sorbents for divalent metal ions with controlled sorption characteristics [17,18,19,20,21]. N-Sulfonatoethylation decreases the sorption ability of chitosan but expands the sorption pH and shifts the optimal pH value toward higher acidities. Such materials exhibit higher sorption rates than standard synthetic ion exchangers do. The degree of sulfonatoethylation of the material changes its sorption ability [22,23].

Artificial gastric juice in environmental models of the stomach can show the impact of gastric juice on probiotic bacteria. The viability of probiotic bacteria is reduced in such stomach models, e.g., by 8.5 logs for *L. casei* and by 11 logs for *B. bifidum* after 2 h [24]. The proteolysis of bacteria by pepsin in the stomach also has an effect on the bacteria’s viability [25].

In this regard, the use of protective shells, as well as transport carriers for the delivery of bacteria into the intestine, seems to be quite justified.

Studies have shown that *B. subtilis* is able to self-coats with biofilm, which could prolong its retention time in the intestine to compared with uncoated *B. subtilis* [26,27,28].

Furthermore, a lipid-membrane-encapsulated oral delivery system for probiotics was constructed by Cao Z. et al. as an IBD treatment [29]. This system has demonstrated that the delivered bacteria could reside in the intestine for 4 h after oral administration. Five days of treatment with lipid-membrane-coated bacteria significantly alleviated the damage to the colonic mucosa and epithelium and the infiltration of immune cells in a murine colitis model [29].

Our results showed that oral delivery using an SEC gel carrier prolonged the residence of test strain bacteria in the animal intestine. The delivery of *B. subtilis 20* using a gel carrier decreased the amount of the test strain in the gut by only two times, compared with a gavage of bacterial suspension that decreased the amount of *B. subtilis* by 200 times. Similar results were established by Khosravi Zanjani MA et al. [30] and Krasaekoopt et al. [31]; they showed that encapsulation in chitosan coatings considerably increased the survival rates of the probiotics.

Despite the *B. subtilis* used in our pilot experiment being stable in acidic environments, it has been shown that the safety of the bacteria of the test strain was increased 100 times as a result of being carried by the SEC gel. A certain contribution to the result could be explained by more effective bacteria adhesion to the intestinal mucosal surface due to prolonged intestinal transit in the gel carrier and a reduced rate of the elimination of probiotic bacteria from the intestine. This gel may be applied in the delivery of other probiotic bacteria or phages that have worse abilities than *B. subtilis* to withstand aggressive external influences, such as hydrochloric acid and gastrointestinal enzymes in the gastrointestinal tract.

The possibility of using gel for the delivery of bacteria in the gastrointestinal tract is also confirmed by our results of in vitro cytotoxicity. In vitro, the gel showed no toxic effect on the cell culture, and when it was used to feed mice we did not observe any adverse reactions in the animals, be they behavioral or allergic.

Our results in vitro of the dye release rate from the gel in an alkaline medium showed that gel demonstrates the first order kinetics, with an initial rapid dose of dye, followed by a slow release and gel degradation in an environment similar to the intestine. The absence of toxic effects on experimental animals after consuming the gel confirmed the safety of its use, since gel degradation and utilization do not lead to the manifestation of intoxication in mice.

In the pilot study, we did not perform a histological assessment of the mice’s tissue after eating the gel, but there others have studied the biocompatibility of sulfated chitosans in vivo. A reparative effect was shown for O,N-(2-sulfoethyl)chitosan on the blood vessel walls of rabbits [32].

Chitosan gel may, therefore, have a therapeutic effect when used as a carrier for bacteria to repair the inflamed intestine in IBD. The results obtained by Ding, K. et al. show that 6-O-sulfated chitosan can act as a supplement aiding cell differentiation [33].

Thus, our results confirmed the possibility of using sodium N-(2-sulfonatoethyl)chitosan gel for the delivery of bacteria into the gastrointestinal tract through the acidic environment in the stomach and for reducing the rate of the elimination of probiotic bacteria from the intestine. The gel was also shown to be non-toxic, and could be used in animals.

## 4. Materials and Methods

### 4.1. Bioresorbable Gel Carrier

**Figure 3:**
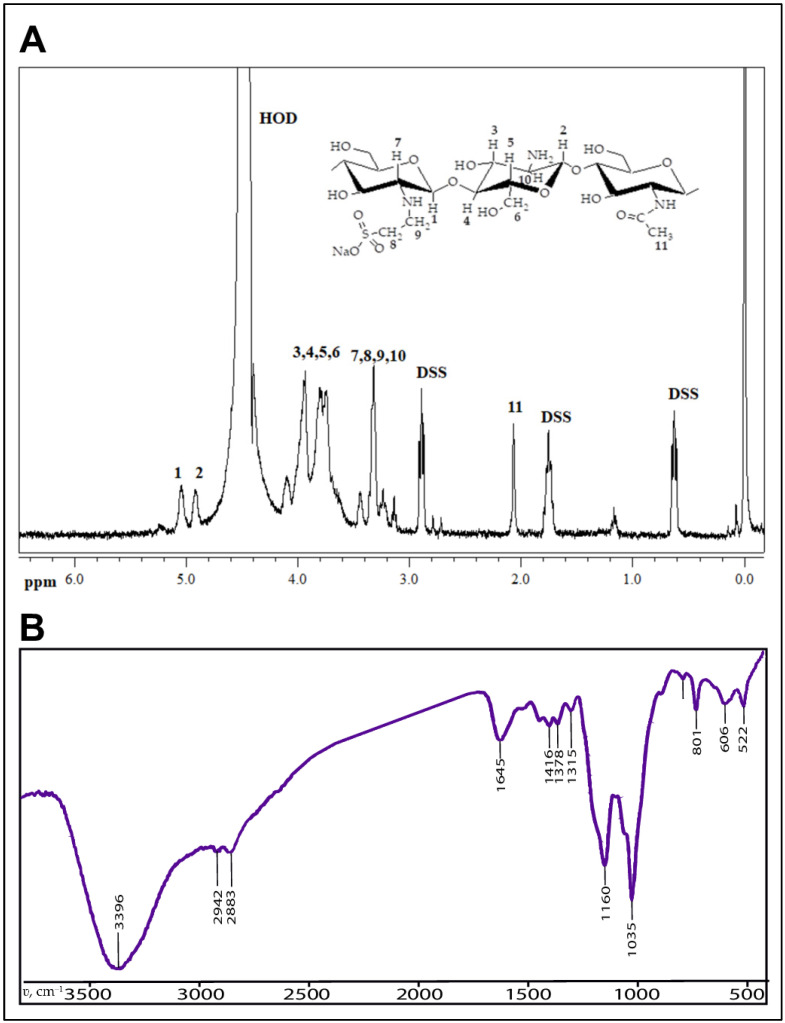
(**A**). The structural units and the 400 MHz ^1^H NMR spectrum of sodium N-(2-sulfonatoethyl)chitosan with degrees of substitution 1.0 (D_2_O/DCl) (DSS–2,2-dimethyl-2-silapentane-5-sulfonate sodium salt was used as an internal ^1^H NMR standard; ppm—parts per million); (**B**). FT-IR spectrum of crosslinked N-(2-sulfoethyl)chitosan with glutaraldehyde.

Samples of sodium N-(2-sulfonatoethyl)chitosan gel crosslinked with glutaraldehyde were synthesized using a method described previously [34]. The composition and structure of the gel were confirmed by elemental analysis using “CHN PE 2400” (Perkin Elmer, Waltham, MA, USA), and FTIR-spectroscopy using a “Spectrum Two” spectrometer (Perkin Elmer, Waltham, MA, USA) (see Figure 3).

All gel samples were sterilized before the experiment, using electron beam sterilization with an absorbed dose 25 kGy [35].

### 4.2. Gel Loading Procedure

Dry sterile gel samples (0.5 g each) under sterile conditions were loaded with a sterile solution of bacteria in a laminar flow hood. Samples were placed in Petri dishes and filled with a PBS solution containing *B. subtilis 20* bacteria in the titer indicated in Table 1. Next, the dishes were stored at +4 °C for 24 h, and then swollen samples were cut into pieces 0.5 × 0.5 cm in size the next day. Each cut sample was transferred into a separate well of a 24-well plate.

**Figure 4:**
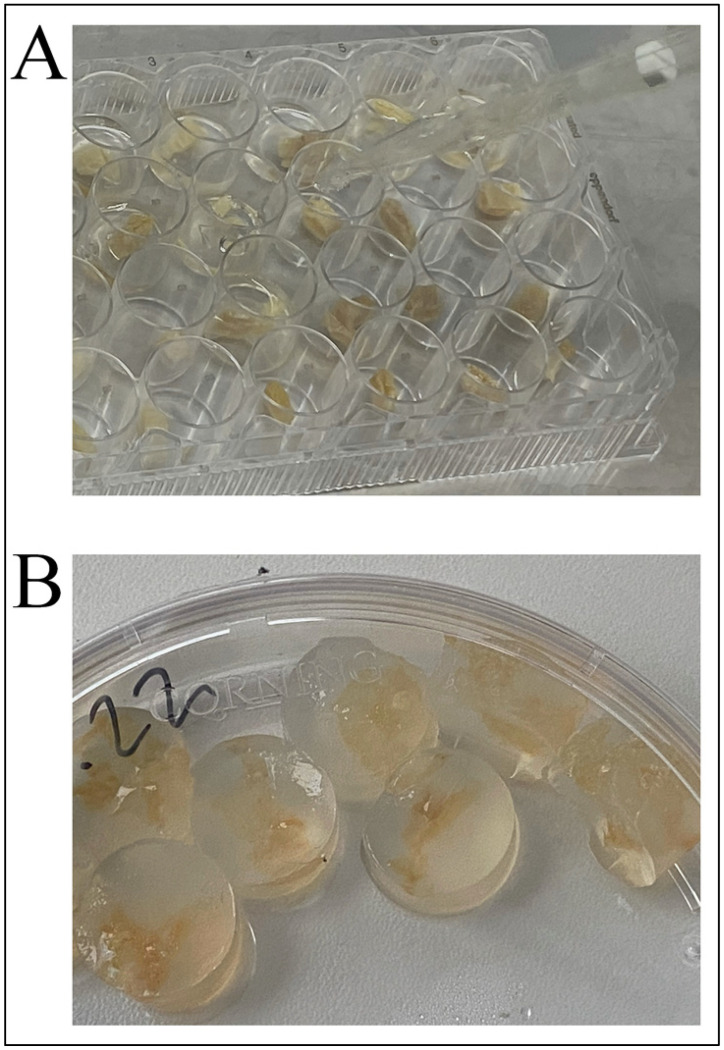
Overview of bacteria-loaded gel samples encapsulated in agar. (**A**). Preparation of gel–agar complexes; (**B**). Final solidified gel–agar complexes.

Then, all bacteria-loaded gel samples were encapsulated in 1% agar (to prevent gel desiccation in air and as a taste modifier for animal feeding) at +37 °C into the same plate wells. After solidification, the resulting gel–agar complexes were removed from the wells (Figure 4), placed in portions (one portion per one feeding) and stored at +4 °C before use.

### 4.3. Animals

Four 14–16 week-old adult CD1 outbred female mice (with confirmed SPF status according to [36]) were housed in individually ventilated cages in a 12/12 h light/dark photoperiod under standard conditions. Food and water were provided ad libitum. Animals were divided into two groups (study and control) in equal proportions, based on feed additives; the study group (n = 2) was fed with standard ration with the test strain-loaded gel carrier samples added once per day for five days, and the control group (n = 2) was fed with standard ration with the test strain bacteria solution added using a gavage.

All the procedures were conducted according to the 3R principle [37] (including small sample size for the pilot experiment), standards of Good Laboratory Practice, institutional Ethics Committee guidelines and the European Convention for the Protection of Vertebrate Animals.

### 4.4. Bacterial Strain and Growth Conditions

The kanamycin-resistant strain *Bacillus subtilis 20* was used to feed the animals. Strain selection for the experiment was substantiated by its ability to grow on selective kanamycin-added medium that allows for precisely calculating CFU for that strain. Bacteria were cultured on plates of Dextrose Casein–peptone agar (Merck, Darmstadt, Germany) at +37 °C for 72–96 h until complete spore formation. Spores of the bacteria were resuspended in sterile phosphate-buffered saline (PBS) solution.

### 4.5. Method of Characterization of Bacillus subtilis 20 Colony Forming Units

The aliquots of the gel (1 g) or mouse feces (100 mg) were placed into 9.0 or 0.9 mL sterile PBS, respectively, and homogenized using a sterile glass rod. A decimal dilution series was prepared in PBS, and 100 µL of the appropriate dilutions were plated on Dextrose Casein–peptone agar containing 100 mg/mL kanamycin. The plates were incubated at +37 °C for 48 h.

### 4.6. Gel Release Model in Different pH Mediums

A pH value equal to 2.2 is equivalent to the stomach pH, and for the intestine pH environment the pH value is equal to 7.4 [38]. The corresponding pH buffers were prepared based on the protocol in [39]. Experimental setup was mounted from polypropylene containers (5 mL), made from 50 mL tubes, and gel placed into the buffer with regard to pH. To maintain the flow around the gel and the required temperature (37 °C), an ImmunoChem-2200 plate shaker-incubator (HTI, North Attleboro, MA, USA) was used.

To detect the release of dye from the gels, spectrophotometry was used. The dye’s (acridoneacetic acid) (Polisan, St. Petersburg, Russia) kinetic release from the gel carrier was visualized based on changes in the optical density of the solution at 355 nm [40].

After loading the gels with dye, the gel samples were washed once in a 1× phosphate-buffered saline (PBS) and placed in the containers (5 mL) with an appropriate pH buffer.

Gel samples prepared for dye release were placed in an appropriate (alkaline or acidic) pH buffer (n = 6 for each medium) and stirred for 2 h at 37 °C.

Every 15 min, 200 μL aliquots of buffer from all containers were sampled, and gels were placed in a new portion of buffer. The optical density of each aliquot was analyzed in black 96-well plates with a translucent bottom on a TriStar LB 941 spectrophotometer (Berthold Technologies, Bad Wildbad, Germany).

### 4.7. Cytotoxicity Evaluation

The effect of the gel on the viability of human fibroblasts was evaluated after 24, 48 and 72 h of incubation with cells (confluent monolayer) in 12-well plates (TPP, Switzerland). Cells were seeded in plates (at least 6 × 10^4^ cells per cm^2^) using the medium, which consisted of DMEM (Gibco, Carlsbad, CA, USA) with 10% fetal serum (StemCell Technologies, Vancouver, Canada), 2 mM L-glutamine (StemCell Technologies, Canada) and combined antibiotics of 100 IU/mL of penicillin and 100 μg/mL of streptomycin (Biolot, St. Petersburg, Russia). The plates were incubated in a CO_2_ incubator at +37 °C in air with 5% CO_2_. After reaching the confluent monolayer, sterile gel samples (approximately 3 mm^3^ in size) were placed on top of the cell monolayer (study group, n = 4). Cultivation of samples with cells was carried out in three replicates.

Fibroblasts cultured without gel samples and any other medium components were used as a positive control (n = 7). Fibroblasts cultured in addition to 10% DMSO (Biolot, St. Petersburg, Russia) were used as negative controls (n = 4). At the end of the incubation period, the samples were removed and the cells were washed with Dulbecco’s phosphate–saline solution (DPBS) (Gibco, Carlsbad, CA, USA). After that, the cells were detached with a 1:1 trypsin: Versen solution (Biolot, St. Petersburg, Russia) and centrifuged for 10 min at 1100 rpm. The pellet was resuspended in the complete medium. Next, the cells were centrifuged for 5 min at 1100 rpm, then washed with 1 mL of PBS. After that, the cell pellet was added to 500 µL of DPBS (Gibco, Carlsbad, CA, USA) containing 100 nM of fluorescent dye Calcein AM Viability Dye (eBioscience Invitrogen, San Diego, CA, USA) for visualization of living cells, followed by incubation for 15 min in the dark at room temperature. Then, 5 µL of fluorescent dye 7-AAD Viability Staining Solution (eBioscience Invitrogen, San Diego, CA, USA) was added to the solution to visualize dead cells and stained for 5 min in the dark at room temperature. For detection and quantitative analysis of living and dead cells, samples stained with the above dyes were analyzed on a FACS Canto II flow cytometer (Becton Dickinson, Franklin Lakes, NJ, USA) using FACS Diva 6.0 software (Becton Dickinson, Franklin Lakes, NJ, USA).

The cell culture was also examined using inverted light microscopy of culture plates to visualize cell growth conditions.

### 4.8. Statistics

Statistical analysis was performed using the IBM SPSS Statistics software (version 25.0) and R software (version 4.1.2). For frequency variables, proportions and confidence intervals were calculated using the method of Wilson E.B. Comparisons were performed by the Pearson Chi-square method or Fisher’s exact method, if applicable (with Bonferroni correction for multiple comparisons). Significance levels are indicated either as absolute values or (in the case of exponential values) as *p* < 0.0001.

## Figures and Tables

**Table 1:** Titer of test strain *B. subtilis 20* in mice feces after gel carrier’s passage through the gastrointestinal tract.

Days of the Experiment	Number of Mice	Titer Value, CFU/g
Bacteria-loaded gel carrier (baseline titer)	1	8.75 × 10^8^
	2	8.75 × 10^8^
Day 0 (before test strain feeding)	1	0.0
	2	0.0
Day 5 after feeding every day with gel carrier additive (end of test strain feeding)	1	4.65 × 10^8^
	2	3.75 × 10^8^
Day 6 (feeding with standard ration only)	1	0.43 × 10^8^
	2	0.89 × 10^8^
Day 7 (feeding with standard ration only)	1	0.10 × 10^8^
	2	0.09 × 10^8^

## Data Availability

Not applicable.
